# Transgenerational plasticity and selection shape the adaptive potential of sticklebacks to salinity change

**DOI:** 10.1111/eva.12688

**Published:** 2018-08-22

**Authors:** Melanie J. Heckwolf, Britta S. Meyer, Talisa Döring, Christophe Eizaguirre, Thorsten B. H. Reusch

**Affiliations:** ^1^ Evolutionary Ecology of Marine Fishes GEOMAR Helmholtz Centre for Ocean Research Kiel Kiel Germany; ^2^ School of Biological and Chemical Sciences Queen Mary University of London London UK

**Keywords:** Baltic Sea, climate change, *Gasterosteus aculeatus*, nonadaptive plasticity, salinity, selection, transgenerational plasticity

## Abstract

In marine climate change research, salinity shifts have been widely overlooked. While widespread desalination effects are expected in higher latitudes, salinity is predicted to increase closer to the equator. We took advantage of the steep salinity gradient of the Baltic Sea as a space‐for‐time design to address effects of salinity change on populations. Additionally, genetic diversity, a prerequisite for adaptive responses, is reduced in Baltic compared to Atlantic populations. On the one hand, adaptive transgenerational plasticity (TGP) might buffer the effects of environmental change, which may be of particular importance under reduced genetic variation. On the other hand, physiological trade‐offs due to environmental stress may hamper parental provisioning to offspring thereby intensifying the impact of climate change across generations (nonadaptive TGP). Here, we studied both hypothesis of adaptive and nonadaptive TGP in the three‐spined stickleback (*Gasterosteus aculeatus*) fish model along the strong salinity gradient of the Baltic Sea in a space‐for‐time experiment. Each population tolerated desalination well, which was not altered by parental exposure to low salinity. Despite a common marine ancestor, populations locally adapted to low salinity lost their ability to cope with fully marine conditions, resulting in lower survival and reduced relative fitness. Negative transgenerational effects were evident in early life stages, but disappeared after selection via mortality occurred during the first 12–30 days posthatch. Modeling various strengths of selection, we showed that nonadaptive transgenerational plasticity accelerated evolution by increasing directional selection within the offspring generation. Qualitatively, when genetic diversity is large, we predict that such effects will facilitate rapid adaptation and population persistence, while below a certain threshold populations suffer a higher risk of local extinction. Overall, our results suggest that transgenerational plasticity and selection are not independent of each other and thereby highlight a current gap in TGP studies.

## 1 INTRODUCTION

Rapid climate change threatens organisms, populations, and species in all ecosystems including the oceans (Poloczanska et al., 2013; Urban, 2015). Whereas marine climate change research mainly focusses on ocean warming and acidification (reviewed in Przeslawski, Byrne, & Mellin, 2015), the effects of salinity shifts on marine populations and ecosystems have rarely been addressed (but see for instance: DeFaveri & Merilä, 2014; Andersson et al., 2015). This oversight is unjustified, as salinity has significant and overriding effects on the physiology of aquatic organisms (Holliday, 1969; Morgan & Iwama, 1991; Muthiga & Szmant, 1987). Models predict that elevated global temperatures will cause increased precipitations and ice melt and thereby rapidly decrease salinity of polar and coastal waters of the North Atlantic region (Gibson & Najjar, 2000; Loder, van der Baaren, & Yashayaev, 2015). Increasing evaporation, on the other hand, is likely to result in elevated salinity in regions around the equator (Boyer, Levitus, Antonov, Locarnini, & Garcia, 2005; Friedman, Reverdin, Khodri, & Gastineau, 2017).

At the organismal level, a rapid change in salinity challenges osmoregulation and the maintenance of plasma ion concentration with depolarized cell membranes inducing apoptosis (Kroemer, Petit, Zamzami, Vayssiere, & Mignotte, 1995). Importantly, ion‐regulation consumes up to 30% of the total energy budget in a cell (Rolfe & Brown, 1997), making acclimation to different salinity regimes possible, but energetically demanding (DeWitt, Sih, & Wilson, 1998). It is therefore not surprising that salinity gradients act as barriers to species range expansion (Larsen, Nielsen, Williams, & Loeschcke, 2008). Consequently, if populations cannot migrate to suitable habitats, they must rapidly adapt and/or acclimate to avoid extinction (Hoffmann & Sgro, 2011).

Recently, transgenerational plasticity (TGP), by which parental environments shape offspring phenotypes, has been proposed as an alternative way to respond to such changes (Mousseau & Fox, 1998; Pigliucci & Müller, 2010). Many different mechanisms might underlie TGP, including physiological, epigenetic, and even cultural inheritance (Laland et al., 2015). Interestingly, these mechanisms can provide a heritable link between environment and phenotype, which might alter the direction of selection and provide an accelerated evolutionary pathway to adaptive solutions (Bossdorf, Richards, & Pigliucci, 2008; Klironomos, Berg, & Collins, 2013). Alternatively, such nongenetic inheritance might buffer effects of natural selection, thereby maintaining neutral and detrimental alleles in the population (Vogl, 1996), which could, at later stages, become beneficial or deleterious under environmental change (Orr & Unckless, 2008).

**Figure 1:**
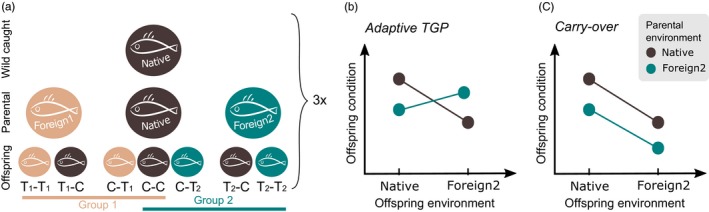
Experimental design and two potential scenarios of transgenerational acclimation. Breeding design conducted three times (a) according to treatment design (Table 1) of wild caught and laboratory bred (parental and offspring) three‐spined sticklebacks from Kiel (KIE, 20 PSU), Thyborøn (THY, 33 PSU) and Nynäshamn (NYN, 6 PSU). The first generation (wild caught) is kept at its native salinity, and the second generation (G1, parental) is exposed to different salinities from the adult stage onwards for five months. The third generation (G2, offspring) is introduced to the respective salinity upon fertilization. Letters refer to control (C, native salinity) and treatment (T, foreign salinity) of parents and offspring, respectively (e.g., T‐C refers to parents in treatment and offspring in control condition). Within the breeding design, group 1 and group 2 were analyzed separately. Assuming the foreign environment of group 2 is challenging, we expect two potential scenarios for group 2 (b, c). Adaptive transgenerational plasticity (TGP) (b) occurs when fitness is highest if environments of parents and offspring match, while carry‐over effects (c) lead to the accumulation of negative effects over generations, leaving offspring of control parents with higher fitness. As foreign environments might also affect offspring condition positively, the sign of the effect could also be reversed

TGP is considered to be adaptive if parental effects act to increase offspring fitness (Mousseau & Fox, 1998), as shown under temperature and acidification stress in fish (Murray, Malvezzi, Gobler, & Baumann, 2014; Shama & Wegner, 2014). In some cases, parental effects lead to a reduction in offspring fitness when parents experienced stressful environmental conditions (Eriksen, Bakken, Espmark, Braastad, & Salte, 2006; Gould, 1988; Marshall, 2008). When a match in parental and offspring environment causes negative effects, for example, via negative carry‐over (Figure 1c), this is considered nonadaptive TGP (Mousseau & Fox, 1998). A recent review found that 41% of transgenerational acclimation experiments led to positive effects, leaving the majority of effects to be negative or neutral (Donelson, Salinas, Munday, & Shama, 2017). Similarly, no overall significant positive effect, but a nonsignificant positive trend, was detected in a comprehensive meta‐analysis of 58 studies in plants and animals, suggesting that TGP is not widespread (Uller, Nakagawa, & English, 2013).

The direction and magnitude of TGP differ not only among species, but also among life stages within a species (Marshall, 2008; Pankhurst & Munday, 2011). Early life stages are particularly vulnerable to environmental changes. For instance, fish larvae lack gills to compensate physiologically for environmental stress (e.g., acidification, salinity change), and most mortality occurs at that stage (Baumann, Talmage, & Gobler, 2012). While it is widely agreed that a better understanding of the interaction of transgenerational plasticity and adaptation is needed (Donelson et al., 2017; Torda et al., 2017), surprisingly few studies have directly accounted for selection in transgenerational studies (but see: Kaufmann, Lenz, Milinski, & Eizaguirre, 2014). A comprehensive framework for TGP studies that acknowledges the bidirectional nature of effects, that is, accelerating via carry‐over vs buffering via adaptive TGP (Figure 1), and their interplay with selection is therefore highly needed.

The Baltic Sea is a semienclosed brackish sea with salinities ranging from approximately 30 to 0 PSU (Practical Salinity Unit). In its central and marginal regions, salinity could decrease of up to 50 % by 2100 (Meier, 2006). Interestingly, recent research shows that genetic diversity is reduced in Baltic populations, due to isolation (DeFaveri, Jonsson, & Merilä, 2013; Johannesson & Andre, 2006) and consequently populations may present reduced adaptive potential in the absence of TGP. Studying the Baltic Sea can therefore serve as a time machine to predict the future of the global oceans (Reusch et al., 2018).

To test for TGP effects across salinity treatments and life stages, we conducted a multigenerational experiment using Baltic Sea three‐spined stickleback (*Gasterosteus aculeatus*) as a model system (Colosimo et al., 2005; DeFaveri & Merilä, 2014). This abundant fish plays important ecosystem roles both as a mesopredator and as a food source (Sieben, Rippen, & Eriksson, 2011). Furthermore, this species is an ecosystem engineer (Harmon et al., 2009) that alters its habitat structure by feeding activity (Anaya‐Rojas et al., 2016). Sticklebacks are also capable of adapting to many environmental conditions including different salinities (Barrett et al., 2011; Colosimo et al., 2005) and exhibit TGP in response to temperature changes over multiple generations (Shama & Wegner, 2014; Shama et al., 2016). Within one generation, all populations of Baltic sticklebacks seem to cope well with decreased salinity while populations native to low‐saline conditions performed poorly under increased salinity conditions (DeFaveri & Merilä, 2014), despite marine ancestors (Makinen, Cano, & Merila, 2006).

Here, we sampled three populations of sticklebacks along a salinity gradient within and at the entrance of the Baltic Sea and exposed them to salinity changes (increased and decreased salinity) in a space‐for‐time experiment (Figure 1a; Table 1). The objectives of this study were (a) to assess whether or not transgenerational acclimation buffers (via adaptive TGP) or accelerates (e.g., via carry‐over) effects of simulated salinity change on fitness‐related traits; (b) to evaluate whether the direction and magnitude of TGP differ between increased salinity and decreased salinity treatments; (c) to investigate whether effects of TGP vary between life stages; and (d) to model *in silico* the contribution of plasticity and selection to observed effects.

**Table 1:** Six experiments conducted using the full factorial breeding design in Figure 1

Location of population origin (native PSU)	Group on Figure 1a	Foreign salinity (PSU)	Salinity treatment	No. of parental families (C, T)	No. of offspring families (CC, CT, TC,TT)	
Nynäshamn (6 PSU)	Group 1	20	Increased	9, 9	6,	6, 7, 7
Nynäshamn (6 PSU)	Group 2	33	Increased	9, 9		6, 4, 3
Kiel (20 PSU)	Group 1	33	Increased	10, 10	6,	6, 6, 6
Kiel (20 PSU)	Group 2	06	Decreased	10, 10		6, 6, 6
Thyborøn (33 PSU)	Group 1	06	Decreased	10, 10	6,	6, 6, 6
Thyborøn (33 PSU)	Group 2	20	Decreased	10, 10		6, 6, 6

Note

Letters refer to treatment conditions (C = control, T = treatment), while the first letter represents the parental conditions and the second letter the offspring conditions.

## 2 METHODS

### 2.1 Experimental design

Baltic three‐spined sticklebacks were collected during the 2014 breeding season in the Kiel (KIE) Fjord, Germany (54°38′N, 10°17′E), at 20 PSU (Practical Salinity Unit). Laboratory bred fish obtained from these wild caught fish will be referred to as “parental generation” (G1) (Figure 1a). This breeding ensures stable salinity conditions for the parental generation. Ten G1 families of 30 individuals each were divided into three treatment groups of 10 fish per family. Each group was kept in 20‐L aquaria connected to a filter tank at 20 PSU water. All laboratory fish were fed *ad libitum* twice daily. At nine months posthatch, we changed salinity from 20 to 6 PSU for one group per family, and from 20 to 33 PSU for another group, while keeping a third control group at 20 PSU. A stepwise acclimation from 20 PSU to the required end level was conducted within 10 days by three PSU steps every second day. The low salinity level (6 PSU) was chosen according to predictions by Meier (2006) and accounting for current salinity fluctuations in Kiel (Federal Maritime and Hydrographic Agency 1986). To assess the effects of global salinity increase and investigate potential trade‐offs of adaptation to low salinity, we also exposed sticklebacks to approximately the same treatment delta but toward increasing salinity (33 PSU). After five months under treatment conditions, six pure crosses within each salinity treatment were performed in vitro*,* which will further be referred to as “offspring generation” (G2). Upon fertilization, clutches were split and separated into different treatments, whereas half was matching and half not matching the saline environment of their parents, that is, offspring from control parents under control and treatment conditions (Figure 1a).

One year later, the same experimental design was repeated using two additional populations: Thyborøn (THY; Denmark, 56°69′N, 8°22′E) and Nynäshamn (NYN; Sweden, 58°90′N, 17°95′E), from high (33 PSU) and low salinity (6 PSU), respectively (Figure 1a, Table 1). We followed the same breeding scheme as for the Kiel population to the exception of fewer families from Nynäshamn (6 PSU, Table 1). We conducted two salinity acclimation experiments per population in parallel and therefore produced the control group (C‐C, Figure 1a) once, resulting in a total of seven treatment groups per population (Figure 1a). We used three populations, each native to a different salinity, and conducted this experimental design (Figure 1a) once per population resulting in 21 different treatments for G2 in total (Table 1).

### 2.2 Fitness measures

To assess the effects of energetically costly osmoregulatory activity, we focused on traits connected to energy storage and growth, as they are impacted by salinity (DeFaveri & Merilä, 2014; Marchinko & Schluter, 2007; Spence et al., 2012). The measured traits are correlated with fitness in fish, including direct (mortality) and indirect fitness measurements (e.g., size, growth, and condition variables; see Wootton 1973; Dufresne, FitzGerald, & Lachance, 1990; Schluter 1995). We sampled the parental generation after 5 months of defined salinity exposure and measured length and weight. Additionally, we assessed the hepatosomatic index (HSI), which is a proxy for energy reserves in form of glycogen storage in the liver (Table 2). Offspring were sampled as eggs, freshly hatched larvae, as well as 12, 30, and 90 days posthatch (dph). Therefore, we measured egg size, yolk sac size to length ratio of fish larvae from pictures taken under a stereomicroscope (Table 2). At 12, 30, and 90 days posthatch, we measured length and weight of the larvae. Additionally, dissections were performed at 30 and 90 dph to assess the HSI of juveniles (Table 2). Crucially, mortality was monitored throughout the experiment to account for possible nonrandom mortality.

**Table 2:** Fitness measures at each sampling time point

Parameter	Age of offspring	Description	Average *N* per treatment group (21)	Average *N* per family within treatment (123)
Egg diameter	5 days postfertilization	Average of 4 diameter measurements per egg	108	18.5
Yolk sac size to length ratio	At day of hatching	Yolk sac area in mm² divided by larvae length	87	15
Standard length (SDL)	12, 30 & 90 dph	Standard length	50, 31 & 56	8.5, 5 & 10
Weight	12, 30 & 90 dph	Weight	50, 31 & 56	8.5, 5 & 10
Hepatosomatic index (HSI)	30 & 90 dph	HSI = (liver weight/total weight) * 100	31 & 56	5 & 10

Note

Fitness‐correlated parameter at each sampling time point with average number of samples per treatment group and per family (crossing) within each treatment group.

### 2.3 Data analyses

We analyzed the effects of parental and offspring treatments on all measurements mentioned above (Table 2). Linear mixed‐effects models were fitted, using *lmer* implemented in the R package “lme4” with Gaussian error and “crossing” as well as “tank” nested within “climate chamber” as random effects. Mortality was analyzed per “tank” as a ratio of “alive” vs “dead” fish, using *glmer* implemented in the R package “lme4” with Binomial error and “crossing” as well as “climate chamber” as random effects. Significance was tested using ANOVA type three, and models were simplified using Akaike information criterion (AIC) (Akaike, 1976) and validated according to the model assumptions. Each population–treatment combination (Figure 1a, Table 1) was analyzed separately, as we were interested in the parallelism of the patterns, that is, how each locally adapted population could respond to a change in salinity. To test for consistency of the patterns across traits and populations, we conducted a meta‐analysis calculating the log response ratio ln*R* (Hedges, Gurevitch, & Curtis, 1999) of each trait within the six experimental groups. Therefore, we averaged the values within each treatment group per tank, crossing and trait (*X*) and divided the treatment average (*X*_T_) by the control average (*X*_C_). This was calculated for all three treatment groups separately (G1Treatment – G2Control (T‐C), G1Control – G2Treatment (C‐T), and G1Treatment – G2Treatment (T‐T); Figure 1a).

$$\text{In}R=\text{In}(\frac{X_{T}}{X_{C}})$$

All traits were weighed equally, as they were all subject to the same study with equivalent levels of replication. All measured response variables are typically positively correlated with Darwinian fitness in fish, such as growth rate, and hepatosomatic index (Table 2). Hence, ln*R* represents increased condition/fitness > 0 and decreased condition/fitness < 0. In particular, we tested for differences in the extent and consistency of TGP between life stages, increased and decreased salinity, populations, and fitness‐correlated traits, such as length, weight, or yolk sac size. We fitted linear models on ln*R* using the function *lm*, tested for significance using ANOVA and conducted a model selection with *stepAIC* and *update* implemented in the R package “MASS.” This model included (a) *population of origin*, (b) *salinity*, (c) *acclimation mode*, (d) *life stage*, and (e) *trait* as well as their interactions as fixed effects prior to model selection (Table 3). We chose 22 dph as a border between early and late life stages as all samples taken after that point possessed all characters of adult sticklebacks, that is, fully developed osmoregulatory organs (Swarup, 1958). To compare between early and late life stages, we additionally ran the same model (excluding the factor *life stage*) for each *life stage* separately. Post hoc tests were carried out using Tukey's “honest significant difference” method *TukeyHSD*. All statistical analyses were run in the R environment (R Core Team 2017).

**Table 3:** Fixed factors used in meta‐analysis and their respective levels

Fixed factor in meta‐analysis	No of levels	Description of levels
Population of origin	3	Nynäshamn from six PSU, Kiel from 20 PSU, Thyborøn from 33 PSU
Salinity	2	Increased salinity (compared to origin), decreased salinity (compared to origin)
Acclimation mode	3	Parents treated and offspring under control condition (T‐C), Parents under control condition and offspring treated (C‐T), Parents and offspring under treatment condition (T‐T)
Life stage	2	Early (before 22 days posthatch), Late (after 22 days posthatch)
Trait	5	Egg size, yolk sac size to length ratio, standard length, total weight, HSI

### 2.4 Accounting for rapid evolution via selection

To assess whether or not observed effects are induced by TGP, we must rule out the effects of selection, for example, against low‐quality offspring (Kaufmann et al., 2014). Therefore, we carried out simulations based on three datasets, by removing samples from treatment groups that had survival rates above a certain threshold. The samples per group have either been chosen (a) randomly (300 replicates, hereafter “random”), or by removing (b) the largest (selecting for small, “small”), or (3) the smallest values (selecting for large, “large”) in each group. Then, we simulated three strengths of selection, reducing the survival rates in the groups that had higher survival than (a) the first quantile (“weak” selection, e.g., reducing survival to 86%), (b) the mean survival rate (“moderate” selection, e.g., reducing survival to 76%), and (c) the third quantile (“strong” selection, e.g., reducing survival to 70%) for each time point individually. After simulating the selection strength, we proceeded with the same analyses as described above for the original dataset, for each of the nine different datasets representing the different forms of selection (weak‐random, weak‐small, weak‐large, moderate‐random, moderate‐small, etc.). In the case that results remained unchanged after considering for selection, we concluded that they are the sole result of TGP. However, in the case that results differed after accounting for selection, they were deemed the product of selection.

## 3 RESULTS

### 3.1 Mortality

**Figure 2:**
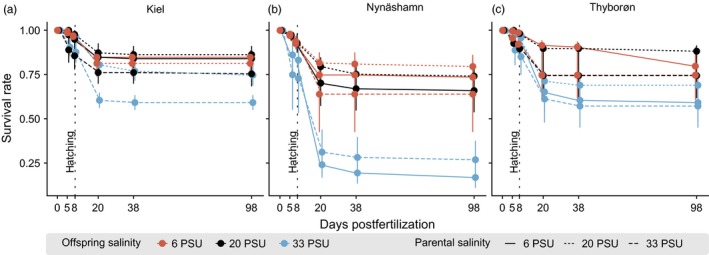
Survival rates throughout the experiment. Survival rate as a ratio of “alive” vs “dead” at different time points postfertilization in days, separately for each population. Time point of hatching was 8 days postfertilization, and early life stages are defined until 30 days postfertilization (22 dph). Colors represent the three different offspring salinity treatments, and the line type denotes the parental salinity treatment

In the parental generation, we observed a significant effect of salinity environment on mortality ($\chi_{2}^{2}$_ _= 7.769, *p *=* *0.021), resulting in slightly lower survival rates at 33 PSU compared to 6 and 20 PSU. In the offspring generation, most mortality occurred after hatching (8 days postfertilization) and before 30 days posthatch (dph). Survival rates were significantly influenced by both offspring and/or parental environment in five of the six population–treatment combinations (Tables 1 and S1), with an increased mortality in increasing salinity treatment (Figure 2). Furthermore, negative effects (i.e., mortality) accumulated over generations via negative carry‐over. One exception was the population from Nynäshamn at 33 PSU, which showed an interaction effect of parental × offspring environment associated with an increased survival rate in a matching environment via adaptive transgenerational plasticity ($\chi_{1}^{2}$ = 19.333, *p *<* *0.001, Figure 2b, Table S1).

### 3.2 Does transgenerational acclimation buffer or accelerate effects of salinity change?

**Figure 3:**
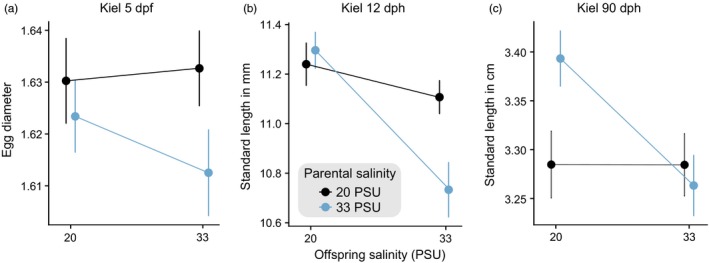
Negative carry‐over effects induced by increased salinity. (a) Egg diameter and standard length at 12 (b) and 90 (c) days posthatch for sticklebacks from Kiel (native salinity = 20 PSU) acclimated to 33 PSU, exemplary for effects induced by increased salinity

Adaptive transgenerational plasticity (TGP) is defined as the interaction between the parental and the offspring salinity environments leading to a positive effect in offspring fitness reaction norms, while nonadaptive TGP decreases offspring fitness reaction norms. Accordingly, we observed two negative interactions of parental and offspring environment in the population from Kiel (20 PSU) at 33 PSU for fish length ($\chi_{1}^{2}$ = 4.481, *p *=* *0.034, Figure 3c) and weight ($\chi_{1}^{2}$ = 7.714, *p *=* *0.005) at 90 dph, resulting in nonadaptive TGP. Specifically, a match of parental and offspring environment led to a decrease in length and weight of the offspring. However, parental salinity environment of 33 PSU led to an increased growth in offspring raised at 20 PSU compared to the control group (parents and offspring at 20 PSU). Additional parental and offspring effects (but not in interaction) were found at all life stages and in all population–treatment combinations. Results are shown in Table S1 (supplementary material). While we found various effects of salinity treatment on offspring, neither the weight ($\chi_{2}^{2}$ = 0.940, *p *=* *0.625) nor the length ($\chi_{2}^{2}$ = 0.829, *p *=* *0.661) or HSI ($\chi_{2}^{2}$ = 0.038, *p *=* *0.981) of the parents was influenced by salinity treatment. Nonetheless, the introduction of G1 parents to a foreign environment (increased and decreased salinity) led to carry‐over effects negatively influencing egg size and yolk sac size to length ratio (6 of 7 effects, e.g., Figure 3a, Table S1). At 12 dph, most of the observed effects (8 of 10 effects) were associated with offspring environments, resulting in size and weight reduction in groups at high salinity but in increased size and weight at lower salinity (Figure 3b). At 30 and 90 dph, most effects were correlated with the parental environment as main effect (16 parental environment significant effects, seven offspring environment, and two interaction effects; Table S1). Contrary to the negative carry‐over effects observed in early life stage, at the adult stage, parental acclimation to foreign salinity (decreased and increased) resulted in a positive fitness enhancing effect.

### 3.3 Meta‐analysis of fitness‐related effects

#### 3.3.1 Does the direction and magnitude of TGP differ between increased and decreased salinity treatments?

**Figure 4:**
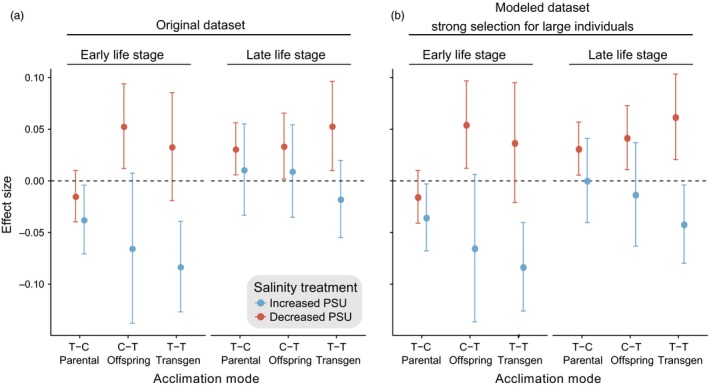
Mean effect size before and after simulating mortality. The effect size of fitness‐related traits for the original dataset (left) and after simulating strong selection for large individuals. The acclimation mode refers to parental treatment (T‐C, parents in treatment, offspring under control condition), offspring treatment (C‐T, parents under control and offspring under treatment condition) and transgenerational acclimation (transgen, T‐T, parents and offspring under treatment condition). Effect size is plotted as mean with 95% confidence interval separately for early and late life stages (before and after 22 dph). The colors indicate the different salinity treatments, respectively, to salinity of origin. Nonoverlap of confidence intervals with the zero line indicates a statistically significant overall effect

Calculating the log response ratio (effect size) ln*R*, we obtained a relative response value that is comparable across traits, populations, and life stages. As the effect size represents a relative measure of fitness‐correlated traits, it can be understood as increased fitness if ln*R *> 0 and decreased fitness if ln*R *< 0. The effect size was significantly influenced by *life stage* (early, late), *trait*, treatment *salinity* (increased, decreased), *population* (Nynäshamn, Kiel, Thyborøn), and the interaction of *salinity* and *treatment mode* (Tables 3 and  4). The population from Nynäshamn had an overall reduced effect size compared to the other populations (*F*_2,144 _= 5.944, *p *=* *0.003, Table 4). Increased salinity resulted in a reduced effect size (i.e., reduced fitness) compared to the decreased salinity treatment (*F*_1,144_ = 32.351, *p *<* *0.001, Figure 4a). This effect was significant when only the offspring (TukeyHSD, *p* adj. = 0.007) or both generations (TukeyHSD, *p* adj. < 0.001) experienced the salinity treatment conditions. However, we could not detect any effect when only the parents were exposed to a different salinity (TukeyHSD, *p* adj. = 0.830, Figure 4a). The interaction of *treatment mode* and *salinity* (*F*_2,144_ = 3.819, *p *=* *0.024, Figure 4a, Table 4) confirmed earlier analyses, namely that negative effects of increased salinity magnified over generations, while positive effects of decreased salinity remained unchanged.

**Table 4:** Results from ANOVA explaining variation in effect size

Fixed factor	*df*	*F* value	*p*
Acclimation Mode	2	0.368	0.693
Life stage	**1**	**14.531**	**<0.001**
Salinity	**1**	**32.351**	**<0.001**
Population	**2**	**5.944**	**0.003**
Trait	**4**	**2.625**	**0.037**
Accl. Mode × Life stage	2	0.327	0.722
Accl. Mode × Salinity	**2**	**3.819**	**0.024**
Accl. Mode × Population	4	0.340	0.850
Life stage × Population	**2**	**3.477**	**0.033**
Life stage × Trait	1	0.731	0.394
Population × Trait	**8**	**3.935**	**<0.001**
Accl. Mode × Life stage × Population	4	2.080	0.0864
Life stage × Population × Trait	**2**	**3.435**	**0.035**
Residuals	144		

Note

Fixed factors are explained in Table 3.

Significant effects are highlighted in bold.

#### 3.3.2 Does the magnitude of TGP differ between life stages?

Late life stages showed an overall larger effect size, corroborating an overall higher condition than early life stages (*F*_1,144_ = 14.531, *p *<* *0.001, Table 4). The significant effect of life stage on effect size encouraged us to split the analyses for each of the life stage.

##### Early life stages

The meta‐analysis on the early life stages subset confirmed the positive effects of decreased salinity and the negative effects of increased salinity treatment (*F*_1,46_ = 39.929, *p *<* *0.001, Table 5) which have been observed in the overall dataset (*F*_1,144_ = 32.351, *p *<* *0.001, Table 4). While transgenerational treatment groups showed a reduced relative fitness (negative effect size) under increased salinity, decreased offspring salinity was associated with increased relative fitness (positive effect size, Figure 4a). Interestingly, salinity and acclimation mode revealed a significant interaction (*F*_2,46_ = 5.392, *p *=* *0.008, Table 5) where transgenerational (T‐T) and developmental (C‐T) acclimation were positive when salinity was decreased but negative when it was increased (T‐T; TukeyHSD, *p* adj. < 0.001, C‐T; TukeyHSD, *p* adj. < 0.001, Figure 4a). No effects were detected when considering only parental treatment (T‐C; TukeyHSD, *p* adj. = 0.926). Comparing among traits, the effect size (ln*R*) was much greater in yolk sac size to length ratio, weight, and length at 12 dph than in egg diameter (*F*_3,46 _= 4.553, *p *=* *0.007), but the direction of the effect (decreased salinity = positive effects, increased salinity = negative effects) remained the same, resulting in an interaction between salinity and trait (*F*_3,46_ = 10.647, *p *<* *0.001).

**Table 5:** Results from ANOVA explaining variation in effect size for subset of “early life stages” and “late life stages”

Effect	Original dataset			Modeled selection Strong selection for large individuals			Modeled selection Strong selection for small individuals			Modeled selection Strong selection random		
	*df*	*F* value	*p*	*df*	*F* value	*p*	*df*	*F* value	*p*	*df*	*F* value	*p*
*Subset “early life stages”*												
Mode	2	0.914	0.408	2	0.826	0.444	2	1.024	0.367	2	0.911	0.409
Salinity	**1**	**39.929**	**<0.001**	**1**	**38.299**	**<0.001**	**1**	**37.559**	**<0.001**	**1**	**40.094**	**<0.001**
Population	2	2.892	0.066	2	1.740	0.187	2	3.188	0.051	2	2.887	0.066
Trait	**3**	**4.553**	**0.007**	**3**	**4.877**	**0.005**	**3**	**4.441**	**0.008**	**3**	**4.562**	**0.007**
Mode × Salinity	**2**	**5.392**	**0.008**	**2**	**5.668**	**0.006**	**2**	**5.125**	**0.010**	**2**	**5.424**	**0.008**
Mode × Trait	6	0.162	0.986	6	0.147	0.989	6	0.178	0.982	6	0.163	0.985
Salinity × Trait	**3**	**10.647**	**<0.001**	**3**	**9.794**	**<0.001**	**3**	**9.619**	**<0.001**	**3**	**10.692**	**<0.001**
Mode × Salinity × Trait	6	1.940	0.094	6	2.066	0.076	6	1.861	0.108	6	1.953	0.092
Residuals	46			46			46			46		
*Subset “late life stages”*												
Mode	**—**	**—**	**—**	2	0.060	0.942	**—**	**—**	**—**	**—**	**—**	**—**
Salinity	**—**	**—**	**—**	**1**	**19.961**	**<0.001**	**—**	**—**	**—**	**—**	**—**	**—**
Population	**2**	**7.349**	**<0.001**	**2**	**5.036**	**0.008**	**2**	**5.145**	**0.007**	**2**	**7.545**	**<0.001**
Trait	2	0.572	0.566	2	1.914	0.308	2	0.564	0.571	2	0.566	0.570
Mode × Salinity	**—**	**—**	**—**	2	2.299	0.106	**—**	**—**	**—**	**—**	**—**	**—**
Salinity × Trait	**—**	**—**	**—**	2	2.649	0.076	**—**	**—**	**—**	**—**	**—**	**—**
Population × Trait	**4**	**2.686**	**0.036**	**—**	**—**	**—**	**4**	**3.058**	**0.020**	**4**	**2.272**	**0.034**
Residuals	99			96			99			99		

Note

Results from ANOVA explaining variation in effect size for subset of “early life stages” and “late life stages” separately. Test statistics for original dataset (actual measurements) and modeled strong selection (survival reduced to 3rd quantile per time point, up to 70%) for “large” (removing the smallest), “small” (removing the largest) and “random selection” (randomly removing individuals, mean values of 300 replicates).

Significant effects are highlighted in bold.

##### Late life stages

The late life stages of fish showed significant variation across populations (*F*_2,99_ = 7.349, *p *<* *0.001) and a population × trait interaction (*F*_4,99_ = 2.686, *p *=* *0.036, Table 5). In particular, we showed that length, weight, and energy reserves (HSI) were significantly lower in the Nynäshamn population (TukeyHSD; NYN‐KIE *p* adj. = 0.001; NYN‐THY, *p* adj.  = 0.029). As populations differed stronger in their energy reserves than in their length or weight, we observed and interaction of population x trait (*F*_4,99_ = 2.686, *p *=* *0.036). Overall, because no transgenerational effects were detectable, and because of high mortality levels at early life stages, it suggests unaccounted effects of selection and nonrandom mortality.

#### 3.3.3 Accounting for rapid evolution via selection

We hypothesized that mortality could alter mean trait distribution in the offspring populations. As it is impossible to run an experiment without selection, we accounted for the classical adaptation process by in silico simulation. To disentangle the effects of TGP and mortality, we simulated selection on early and late life stages and repeated the statistical models presented in Table 5. As mortality was comparably low at early time points and selection strength was calculated according to 1st, mean and 3rd quantile of survival rate separately for each time point (weak, moderate, and strong selection, respectively), the effects observed in the early life stages remained stable throughout all selection strengths and directions (small, large, random; Table 5). Most mortality occurred at the end of the early life stage. In the late life stage, the negative effects of increased salinity treatment vanished. By selecting for large individuals at late life stages, we almost entirely recreated the negative effect of increased salinity (weak selection: *dismissed during model selection*, median selection: *F*_1,96_ = 14.006, *p < *0.001, strong selection: *F*_1,96_ = 19.961, *p *<* *0.001). Even though the interaction of acclimation mode x salinity could not be recreated (weak selection: *dismissed during model selection*, median selection: *F*_2,96_ = 1.989, *p = *0.142, strong selection: *F*_2,96_ = 2.299, *p *=* *0.106), the effect became stronger with increasing selection strength and the transgenerational treatment group (T‐T) was significantly different from the control (Figure 4b). But when we selected randomly or for small individuals, the effects observed in the original dataset at late life stages did not change, no matter the selection strength (Table 5). As effects obtained by our selection model in the early life stages and the original dataset did not differ, we can conclude that not selection but plasticity was shaping these responses, because we controlled the genetic background by a split‐clutch design. However, the negative effects of increased salinity that vanished in the late life stages could be recreated by selecting for large individuals in the low mortality groups. This suggests that our selection model leveled out selection against poor quality offspring in the high mortality groups that might have naturally occurred throughout our experiment.

## 4 DISCUSSION

For about a decade, ocean acidification and warming have been in the focus of evolutionary ecology research, while changes in salinity regime in large ocean areas, due to altered precipitation patterns and melting glaciers, have received relatively little attention (Friedman et al., 2017; Loder et al., 2015; Przeslawski et al., 2015). Given the metabolic costs of osmoregulation to all marine life, it is important to understand the effects of salinity change on species survival and evolutionary potential. In the Baltic Sea stickleback, populations are locally adapted to their saline conditions (DeFaveri & Merilä, 2014; DeFaveri et al., 2013; Guo, DeFaveri, Sotelo, Nair, & Merilä, 2015). We showed that, for populations originating from low salinities, their natural local adaptation resulted in the loss of the ability to cope with fully marine conditions. This was particularly evident from the low survival rates and poor condition of fish acclimated to increased salinity over two generations. Increased salinity reduced fitness‐correlated traits of the early life stages in the mid‐ and low‐saline populations (Kiel and Nynäshamn), while no effects were detected in the late life stages (Figure 4a). Here, nonadaptive transgenerational plasticity resulted in an accumulation of negative effects via negative carry‐over at increased salinity. On the other hand, sticklebacks from all populations were capable of acclimating to desalination, as predicted for many coastal regions of the northern hemisphere (Gibson & Najjar, 2000; Meier, 2006). Interestingly, survival rates even increased in the marine population (Thyborøn) under experimental desalination. While this pattern appears surprising, it is in line with previous studies on Baltic and marine sticklebacks (DeFaveri & Merilä, 2014; Marchinko & Schluter, 2007) and can most likely be assigned to the fact that approximately 11 PSU is isosmotic to the body fluids of sticklebacks (Schaarschmidt, Meyer, & Jürss, 1999). Furthermore, decreasing salinity led to an increase in fitness‐correlated variables, such as length, weight, or yolk sac size to length ratio. These effects remained unchanged by transgenerational exposure to low‐saline conditions, demonstrating no specific effects of TGP. As the high salinity treatment (33 PSU) was further away from the physiological isosmotic level than the low salinity treatment (6 PSU), it seems likely that osmoregulation in full‐marine environment demanded more energy than in six PSU, typically found in the central and northern Baltic Sea. Furthermore, it has been shown that osmoregulatory plasticity, in terms of kidney morphology and gene expression, is reduced in low‐saline compared to a high‐saline Baltic sticklebacks (Hasan et al., 2017).

To date, experimental studies are inconclusive as to whether transgenerational effects accelerate or buffer the effects of environmental change (Donelson et al., 2017; Uller et al., 2013). Our results demonstrate that the direction of TGP effects cannot be generalized as buffering or accelerating, and reveal to be context‐dependent (i.e., life stages and direction of salinity change). Furthermore, not only the environmental shift per se, but also the environmental variability seems to play an important role in the extent of TGP (Shama, 2017). As a result, we hypothesized that the direction (accelerating/buffering) and the magnitude of transgenerational plasticity differ between these more (increased salinity) and less (decreased salinity) stressful treatments.

Using a meta‐analysis approach, we tested for consistency, magnitude, and direction of transgenerational plasticity among populations and traits in the face of two different salinity change scenarios. First, confirming local adaptation, we found strong population differences. Second, the direction of salinity change (increased or decreased) altered significantly the consistency, magnitude, and direction of transgenerational effects on the offspring's traits reaction norm (Figure 4a). In particular, a transgenerational increase in salinity resulted in a cumulative negative effect associated with a further decrease in fitness‐correlated traits across early life stages, which is considered nonadaptive TGP. Such negative carry‐over effects could result from the costs of osmoregulation against a steep osmotic gradient combined with a trade‐off in parental provisioning. They may also result from the alteration of sperm quality of males as previously reported after infection experiment in sticklebacks (Kaufmann et al., 2014). The allocation of resources between reproduction and growth shapes population dynamics by affecting adult survival, reproductive output, and offspring survival (Schwagmeyer & Mock, 2008). If a shift in resource allocation under unfavorable conditions with low chances of offspring survival can ensure survival of the parental generation, this can ultimately enhance population persistence in species that reproduce through repeated discrete clutches (Hoffmann & Merilä, 1999; Kozłowski & Wiegert, 1986). In contrast, when salinity decreased relative to the habitat of origin, the offspring response was largely positive and associated with increased fitness‐correlated effect size. Importantly, this response was independent of transgenerational acclimation suggestive of relaxed evolutionary pressure in more favorable conditions.

We hypothesized that TGP may vary between early and late life stages, because different life stages are differently susceptible to stress (Baumann et al., 2012). We confirmed that early life stages are particularly vulnerable to increased salinity. While we found negative carry‐over effects of transgenerational acclimation in the early life stages, these effects vanished in late life stages. One possible hypothetical explanation is that developmental plasticity takes time to adjust phenotypes to an optimum state. The distance of treatment to isosmotic conditions, which is higher under increased salinity, might be of particular importance. One would therefore predict that small changes are easier to handle than larger changes for larvae and juvenile fish which do not have fully developed primary osmoregulatory organs (Swarup, 1958). On the other hand, traits exhibiting nonadaptive plasticity might ultimately be under stronger selection than traits closer to the phenotypic optimum (Ghalambor et al., 2015), and thereby, the recovery of the late life stages could be the result of selection.

We hypothesized that selection could reduce negative carry‐over effects by removing individuals further away from the phenotypic optimum, as observed in the late life stages after most mortality occurred. Rapid adaptive evolution via selection occurs within few generations (De Wit, Dupont, & Thor, 2016; Eizaguirre, Lenz, Kalbe, & Milinski, 2012) and even within a single generation, owing to classical adaptive processes (Hendry & Kinnison, 1999; Schoener, 2011). To disentangle selection from plastic acclimation effects, we modeled different directions and strengths of selection to control for mortality. Our results suggest that nonadaptive transgenerational plasticity in conjunction with selection can shift existing phenotypic diversity toward the optimum phenotype, here the control phenotype, and thereby accelerates evolution. Many, mainly theoretical approaches, predict that adaptive plasticity accelerates adaptive evolution by genetic assimilation (Bossdorf et al., 2008; Laland et al., 2015; Waddington, 1953). However, there is evidence that nonadaptive plasticity is also capable of potentiating rapid adaptive evolution of gene expression. For example, a guppy transplant experiment found that the most plastic transcripts evolved reduced plasticity due to strong selection against nonadaptive plasticity (Ghalambor et al., 2015). However, if environmental change exceeds a critical rate, plasticity alone is unlikely to facilitate species persistence (Chevin, Lande, & Mace, 2010). From long‐term field observations, we know that even if a population evolves in response to rapid climate change, this does not guarantee population persistence (Nussey, Postma, Gienapp, & Visser, 2005). In particular, a study on great tits and prey availability showed that despite increased plasticity and genetic changes the overall reproductive success continued to decline (Nussey et al., 2005). It is beyond the scope of this study to assess whether along with the shift in phenotypic traits, selection also altered the underlying genetic diversity. However, if this was the case, this might have two potential outcomes: (a) Nonadaptive transgenerational plasticity increases directional selection and therefore accelerates evolution toward an adaptive solution or (b) nonadaptive transgenerational plasticity magnifies the effects of environmental change and increased selection pressure leads to extinction at a higher rate as predicted from within generation acclimation experiments.

## 5 CONCLUSION

Our study demonstrates that TGP is context‐dependent. It interacts with selection and is overall of negative value the further away the environment shifts traits from their optimum. To make correct inferences on TGP, the importance of integrating mortality effects into the analysis of transgenerational experiments cannot be overemphasized. As hypothesized, selection occurring within one generation changed the outcome of transgenerational experiments, and selection processes were altered by nonadaptive transgenerational plasticity. Specifically, due to negative carry‐over effects, the offspring phenotype was moved further away from the local optimum, here the control phenotype, and thereby nonadaptive TGP indirectly increased selection pressure. If this ultimately facilitates rapid adaptive evolution and population persistence or leads to extinction by reducing genetic variation, and population size remains to be investigated. To fully resolve the interaction of genetic adaptation and (transgenerational) plasticity, underlying shifts in genetic diversity and levels of plasticity need to be identified for each generation.

One salient finding of our study was that even in a single species the direction and magnitude of TGP depended highly on the particular environmental factor in combination with life stage. Instead of the current generalization of the buffering nature of TGP, we demonstrated an approach that can tease apart the various effects of TGP by applying a meta‐analysis and modeling selection. Ultimately, this provides a tool to investigate the interplay of plasticity and selection in response to environmental change, which is crucial for understanding the evolutionary potential of marine populations.

## CONFLICT OF INTEREST

None declared.

## ETHICAL STATEMENT

This study was conducted in line with German animal welfare standards (MELUND number: V 312‐7224.121‐19), and the authors have no conflict of interest to declare.

## DATA ARCHIVING

Raw data to this study on measurement of fitness‐correlated factors, such as mortality, clutch size, or weight, are available at PANGAEA (https://doi.org/10.1594/pangaea.892493).
