# Supplementary material for: Transgenerational plasticity and selection shape the adaptive potential of sticklebacks to salinity change
Source: Evol Appl. 2018 Aug 22;11(10):1873–85. doi: 10.1111/eva.12688 (PMC6231470; doi:10.1111/eva.12688)
Supplement: Supplementary file 1 [file EVA-11-1873-s001.docx]

**Supplementary Material**

**Table S1 Results from linear mixed models for each of the six experiments**

|  |  | Nynäshamn  (6 --> 20 PSU) | | | Nynäshamn  (6 --> 33 PSU) | | | Kiel  (20 --> 06 PSU) | | | Kiel  (20 --> 33 PSU) | | | Thyborøn  (33 --> 6 PSU) | | | Thyborøn  (33 --> 20 PSU) | | |
| --- | --- | --- | --- | --- | --- | --- | --- | --- | --- | --- | --- | --- | --- | --- | --- | --- | --- | --- | --- |
| Response | fixed factor | Chisq | Df | *P* | Chisq | Df | *P* | Chisq | Df | *P* | Chisq | Df | *P* | Chisq | Df | *P* | Chisq | Df | *P* |
| egg diameter | female weight | 5.819 | 1 | 0.016 | 8.748 | 1 | 0.003 | 6.085 | 1 | 0.014 |  |  |  | 17.168 | 1 | <0.001 | 9.124 | 1 | 0.003 |
|  | offspring PSU |  |  |  |  |  |  | 5.379 | 1 | 0.020 |  |  |  |  |  |  |  |  |  |
|  | parental PSU | 4.978 | 1 | 0.026 | 9.877 | 1 | 0.002 | 35.917 | 1 | <0.001 | 7.994 | 1 | 0.005 |  |  |  | 5.496 | 1 | 0.019 |
|  | offspring x parental PSU |  |  |  |  |  |  |  |  |  |  |  |  |  |  |  |  |  |  |
| yolk-sac to length ratio | offspring PSU |  |  |  |  |  |  |  |  |  |  |  |  |  |  |  |  |  |  |
|  | parental PSU |  |  |  |  |  |  |  |  |  |  |  |  |  |  |  | 8.702 | 1 | 0.003 |
|  | offspring x parental PSU |  |  |  |  |  |  |  |  |  |  |  |  |  |  |  |  |  |  |
| SDL 12dph | offspring PSU |  |  |  | 7.294 | 1 | 0.007 | 11.097 | 1 | <0.001 | 0.039 | 1 | 0.844 | 8.093 | 1 | 0.004 | 5.100 | 1 | 0.024 |
|  | parental PSU | 4.237 | 1 | 0.040 |  |  |  |  |  |  | 0.054 | 1 | 0.817 |  |  |  |  |  |  |
|  | offspring x parental PSU |  |  |  |  |  |  |  |  |  | 3.166 | 1 | 0.075 |  |  |  |  |  |  |
| weight 12dph | offspring PSU |  |  |  | 9.691 | 1 | 0.002 | 9.068 | 1 | 0.003 | 2.938 | 1 | 0.087 | 12.131 | 1 | <0.001 | 14.79 | 1 | <0.001 |
|  | parental PSU | 5.101 | 1 | 0.024 |  |  |  |  |  |  |  |  |  |  |  |  |  |  |  |
|  | offspring x parental PSU |  |  |  |  |  |  |  |  |  |  |  |  |  |  |  |  |  |  |
| SDL 30dph | offspring PSU |  |  |  |  |  |  |  |  |  |  |  |  |  |  |  |  |  |  |
|  | parental PSU |  |  |  |  |  |  | 13.142 | 1 | <0.001 |  |  |  |  |  |  |  |  |  |
|  | offspring x parental PSU |  |  |  |  |  |  |  |  |  |  |  |  |  |  |  |  |  |  |
| weight 30dph | offspring PSU |  |  |  |  |  |  | 4.712 | 1 | 0.030 |  |  |  |  |  |  |  |  |  |
|  | parental PSU | 6.234 | 1 | 0.013 | 4.995 | 1 | 0.025 | 21.572 | 1 | <0.001 | 2.712 | 1 | 0.100 |  |  |  |  |  |  |
|  | offspring x parental PSU |  |  |  |  |  |  |  |  |  |  |  |  |  |  |  |  |  |  |
| hsi 30dph | offspring PSU |  |  |  | 3.112 | 1 | 0.078 | 4.375 | 1 | 0.036 |  |  |  |  |  |  |  |  |  |
|  | parental PSU |  |  |  | 7.286 | 1 | 0.007 |  |  |  |  |  |  | 6.332 | 1 | 0.012 |  |  |  |
|  | offspring x parental PSU |  |  |  |  |  |  |  |  |  |  |  |  |  |  |  |  |  |  |
| SDL 90dph | offspring PSU |  |  |  |  |  |  | 0.138 | 1 | 0.71 | 0.005 | 1 | 0.946 | 4.106 | 1 | 0.043 | 2.592 | 1 | 0.107 |
|  | parental PSU |  |  |  |  |  |  | 6.133 | 1 | 0.013 | 11.69 | 1 | <0.001 |  |  |  | 2.365 | 1 | 0.124 |
|  | offspring x parental PSU |  |  |  |  |  |  | 0.604 | 1 | 0.44 | 4.481 | 1 | 0.034 |  |  |  |  |  |  |
| weight 90dph | offspring PSU |  |  |  |  |  |  |  |  |  | 0.139 | 1 | 0.71 | 4.705 | 1 | 0.03 |  |  |  |
|  | parental PSU |  |  |  |  |  |  | 15.078 | 1 | <0.001 | 11.818 | 1 | <0.001 |  |  |  |  |  |  |
|  | offspring x parental PSU |  |  |  |  |  |  |  |  |  | 7.714 | 1 | 0.005 |  |  |  |  |  |  |
| hsi 90dph | offspring PSU |  |  |  | 4.354 | 1 | 0.037 | 9.609 | 1 | 0.002 | 1.275 | 1 | 0.259 | 7.437 | 1 | 0.006 | 2.049 | 1 | 0.152 |
|  | parental PSU |  |  |  |  |  |  | 3.045 | 1 | 0.081 | 12.563 | 1 | <0.001 | 5.057 | 1 | 0.025 |  |  |  |
|  | offspring x parental PSU |  |  |  |  |  |  |  |  |  | 3.166 | 1 | 0.075 |  |  |  |  |  |  |
| Mortality 90dph | offspring PSU | 11.625 | 1 | <0.001 | 163.857 | 1 | <0.001 |  |  |  | 16.308 | 1 | <0.001 | 13.741 | 1 | <0.001 | 43.865 | 1 | <0.001 |
|  | parental PSU | 2.940 | 1 | 0.086 | 24.116 | 1 | <0.001 |  |  |  | 24.567 | 1 | <0.001 | 12.346 | 1 | <0.001 |  |  |  |
|  | offspring x parental PSU |  |  |  | 19.333 | 1 | <0001 |  |  |  |  |  |  | 3.867 | 1 | 0.049 |  |  |  |

*Results from linear mixed models for egg size. yolk-sac to length ratio. SDL. weight and HSI separately for each of the six experiments. Models were tested with all fixed factors and simplified according to AIC. Factors not reported were dropped during model selection and can therefore be considered non-significant*
